# You read best what you read most: An eye tracking study

**DOI:** 10.16910/jemr.13.2.9

**Published:** 2020-11-05

**Authors:** Uroš Nedeljković, Kata Jovančić, Nace Pušnik

**Affiliations:** University of Novi Sad, Serbia; University of Ljubljana, Slovenia

**Keywords:** font tuning effect, familiarity, typography, eye tracking, region of interest, reading, art perception

## Abstract

At the threshold of the digital era, Zuzana Licko was of the opinion that familiar letterforms owe legibility to centuries-long exposure and that all new, prototypically unmatching forms would be equally legible if used as frequently. This paper examined the legibility in the context of familiarity – is it affected by the time of exposure to a particular typeface or a typeface’s universal structure. We ran repeated measures tests with exposure period in-between. The experiment was conducted using for this purpose designed typefaces as stimuli, and the eye-tracking on-screen reading technology. The results confirmed that one’s familiarity with a typeface influences one’s reading speed. The universal letter structure, recognised by Frutiger as the prototype skeleton, is the constant that a priori provides legibility. On the other hand, the period of exposure to uncommon letterforms also has a positive impact on legibility. Therefore, considering that the period of familiarity with the humanist letterforms has been continuous since their establishment, the maxim from the dawn of the digital era can be regarded as valid.

## Introduction

For centuries of practice, typography was based on and examined through the lens of implicit knowledge. However, at the beginning of the 20th century, in pursuit of modern typographic forms, which like all the other arts and crafts had to deviate from tradition, implicit knowledge came into collision with the doctrine which had defined the framework for educational centres at the international level. The effects of modernism, visible at the end of the last century, further inspired individual empirical projects. These yielded results which only raised numerous new questions for the educators and researches, who today can finally distinguish between implicit and explicit knowledge. The relationship between the typeface form and reading comfort, that is, how the former affects the latter, first came into focus when the avant-garde challenged the traditional principles, and then again, at the beginning of the postmodern era. These questions interested not only the practitioners and theoreticians of typography, but researchers in other areas and disciplines as well.

The reduction of form and content in the Kleinschreibung system, i.e. the orthographic-typographic reform of the Bauhaus, inevitably points to the functionality of unicase, primarily questioning the legibility and categorically dismissing the versals (capital letters) as non-utilitarian. When we, however, look at the literature on typeface legibility, we can see that this idea requires empirical testing since the recent findings, among others, confirm the effects of familiarity in the context of typeface legibility (1, 2, 3). Furthermore, the postmodernist maxims of Peter Martens, Jeffery Keedy, and Zuzana Licko: “Illegibility does not exist” (4); “Those are all conventions” (5); “You read best what you read the most” (6), are all calling for empirical research that will prove that legibility depends on familiarity. At the threshold of the digital era, these designers claimed that some typefaces were more legible than others due to their familiarity, i.e. the exposure to a typeface form over many decades or even centuries. Based on these premises and the empirical research on typeface legibility, we examined the nature of familiarity – is it affected by the time of exposure to a particular typeface or a typeface’s universal structure.

In other words, this study aims to check whether the font tuning effect is dependent on the familiarity with typefaces’ common skeleton or rather familiarity with specific typeface characteristics. The experiment was carried out using three fonts for each of the defined familiarity levels, which are based on conditions of previous exposure and common traits they share with the prototypical skeleton. Therefore, one widely known-common typeface was used, and two new ones (one with common and the other with uncommon skeleton) which had been designed for this purpose. The authors conducted the repeated measures test for on-screen reading with the period of exposure in-between for each of the three typefaces and analysed the total fixation duration.

The prototypical typeface concept and the historical development of experimental unicase typography are described in the following section, to explain the background of the research question.

## Historical background

### Type standardisation in the Western world

The need to have a uniform type, so as to make communication easier and texts more understandable, first arose during Humanism. The first attempt of type standardisation is the transition to the Roman type. During the Renaissance, the increased demand for books led to the development of new typefaces. There was, however, little difference between them. The general admiration for antiquity and what befitted man resulted in many Renaissance scholars and artists (Felice Feliciano, Luca de Pacioli, Geofroy Tory, Albrecht Dürer) turning to and independently studying the Roman square capitals, also called capitalis monumentalis. The differences between the authors’ works lay in the proportions – the number of the squares in the letters’ height, i.e. the raster density under the common constant – the square.

**Figure 1.**
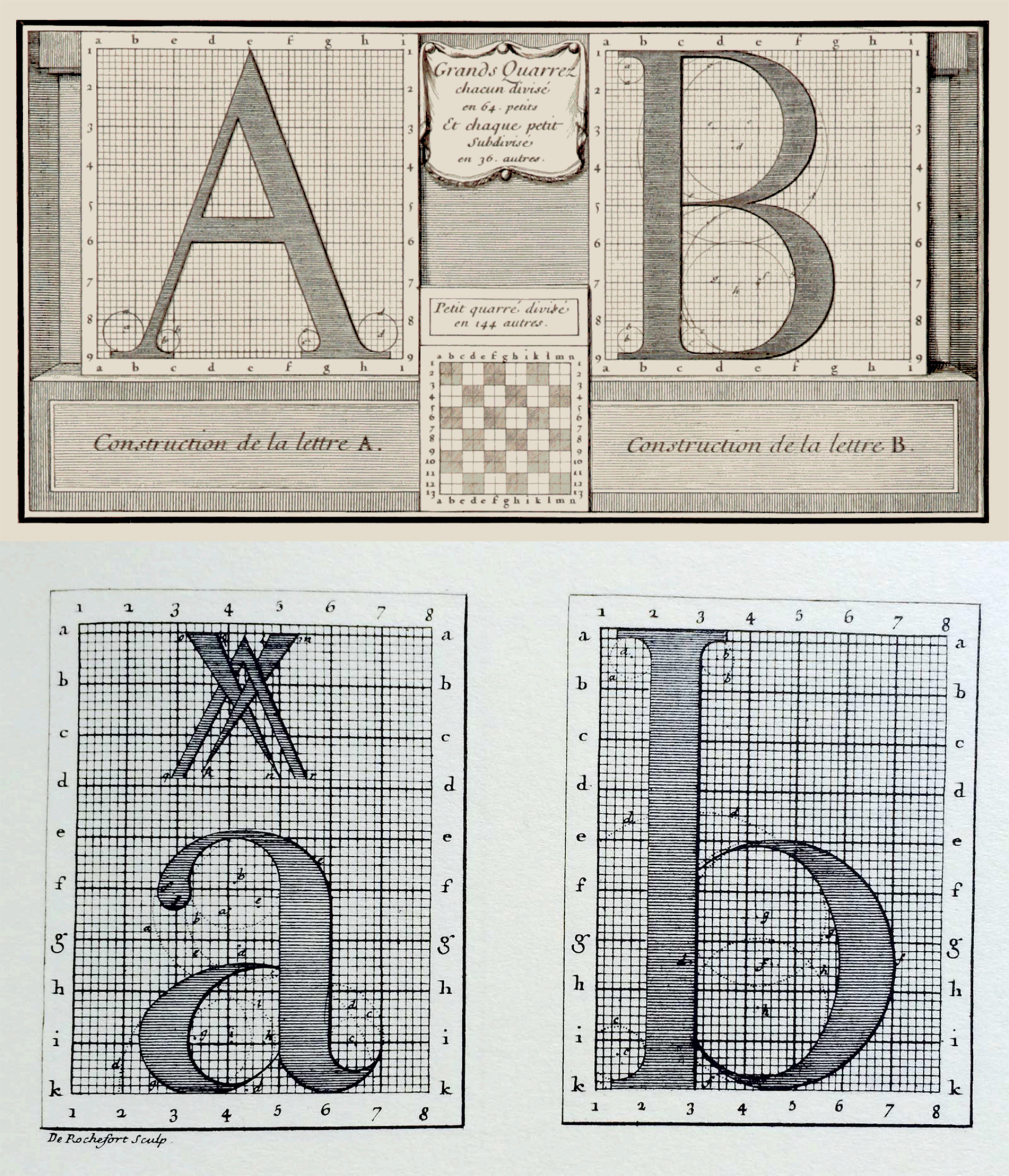
Roman du Roi, uppercace and lowercase construction.

Pioneers in the field, masters Nicholas Jenson and Francesco Griffo, used the proportions of Roman square capitals to shape and cut the letters of the first Roman typefaces. The main challenge they, and other Roman typeface designers, were faced with was how to align the Humanistic minuscule with the Roman square capitals. For, whereas the latter, angular and geometric, was based on the Greek capitals, the former was developed from handwritten letterforms, making the harmonisation of these two an issue to be solved through the coming centuries and a succession of different Roman type designs. Developed in 1698, Romain du Roi (French: “King’s Roman”) typeface, served as a breakthrough, of a kind, by managing to successfully bring the majuscule and miniscule letters together. It became a model of new proportions, where the lowercase letters were appended to the uppercase ones by being systematically constructed on the same principles (Figure 1).

**Figure 2.**
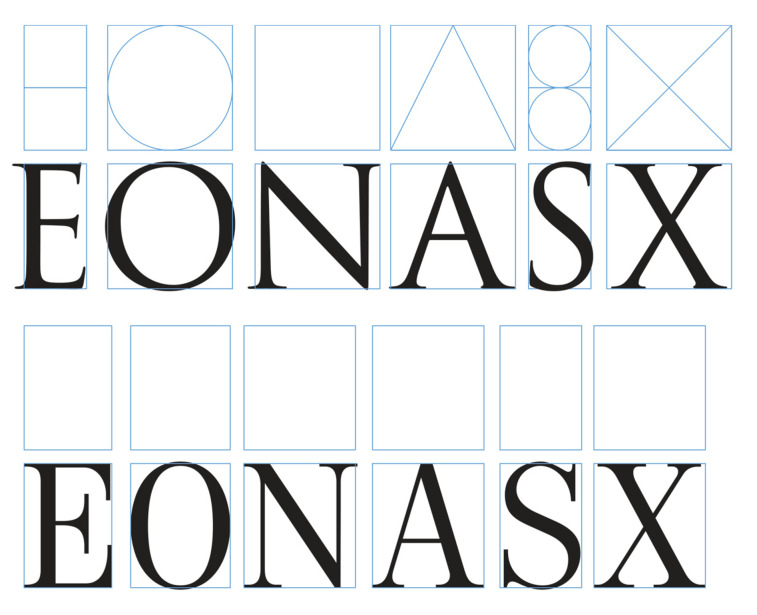
Classical and ‘Modern’ letter proportions (adapted from ( 7)). The typeface ‘Trajan’ by Carol Twombly (up) and the typeface ‘Neoplanta BG’ by Stjepan Fileki (down).

The first steps toward the modernisation of the Roman typeface design had to do with the changes to the proportions of the characters. The wide letters, which, like those they had been based on – capitalis monumentalis, filled a square, i.e. an inscribed circle, were narrowed, while the Humanist typeface letters, defined by a half-square, were widened (Figure 2). Additionally, the axis of the letters was also altered, so it became perpendicular to the baseline, something that could already be found in the Transitional typefaces, e.g. Baskerville. The other stylistic characteristics were over time subjected to change as well, in accordance with the advancement of graphic technology and materials. The appearance of the satin paper, for instance, enabled the typographers of the time to print fine lines and make delicate contrasts in small gradations. John Baskerville’s Roman typeface is the first example of this kind, since he himself experimented with both paper and ink production. Giambattista Bodoni used Baskerville’s light form as a role model for his Roman “Bodoni” in 1785, while Firmin Didot achieved extreme contrast in thick and thin strokes in his types, around 1783. Both Bodoni and Didot are considered the fathers of the so-called “Modern” Roman forms.

The Industrial Revolution, which lasted until mid-1800s, brought on the rise of cities and created the need for mass media and advertising. Thus, the type foundries began manufacturing various decorated and shaded Roman typefaces. It was, however, Vincent Figgins’ Roman, from 1815, distinguished by its lack of elegant contrast, which answered the need.

**Figure 3.**
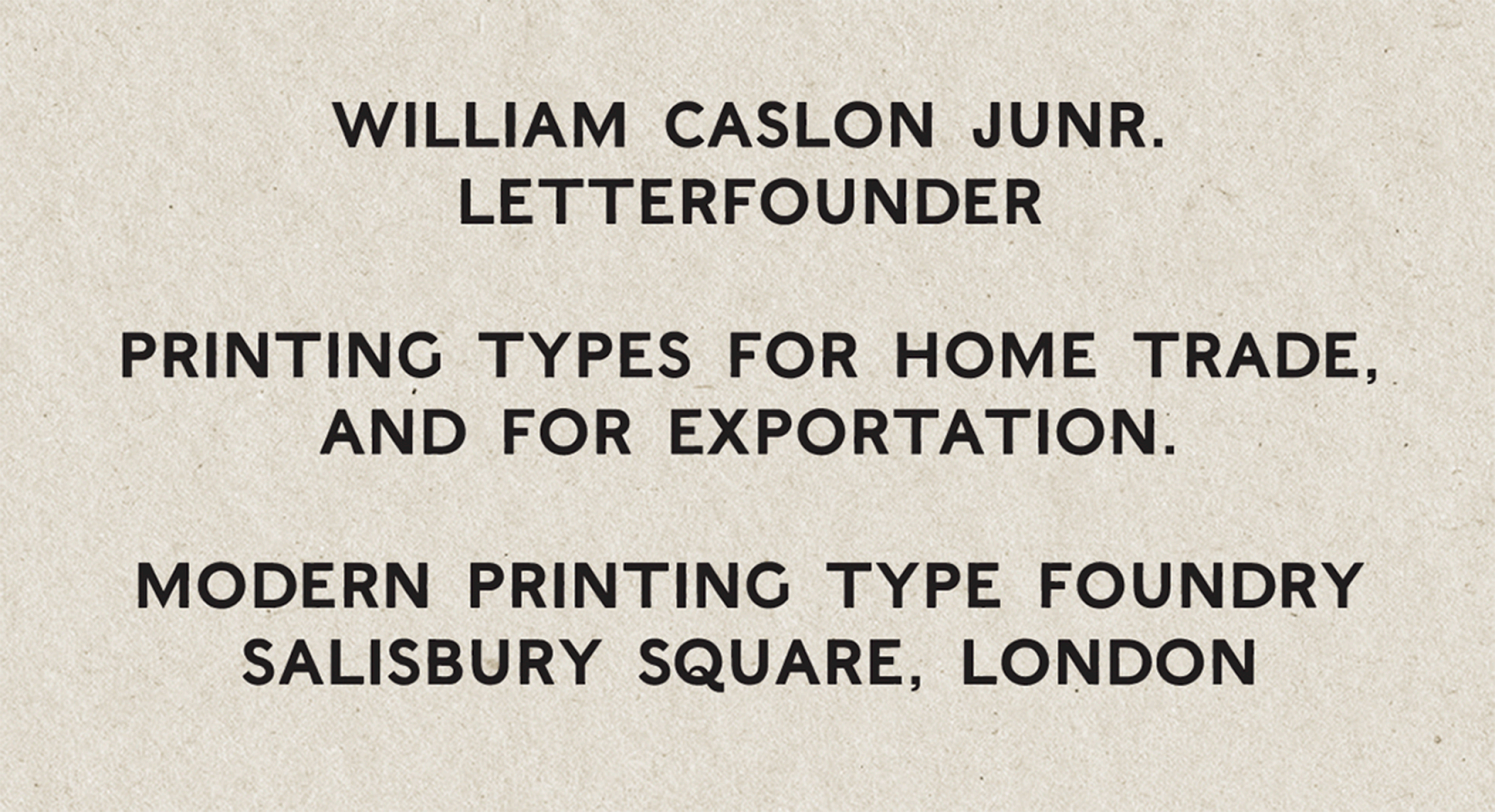
The typeface ‘English Egyptian’ by William Caslon Junior, digitalised.

Similar types, called “Egyptian”, were becoming more frequent. Only a year later, in 1816, William Caslon Junior promoted his “English Egyptian” – the first sans-serif typeface (Figure 3). Its appearance initiated a series of similar sans-serifs from other type foundries. That is how the first forms of the so-called Grotesque typefaces were developed, which evolved into Neo-Grotesque (at the end of the 19th century), then Geometric (ca. 1925) and Humanist (cca. 1930).

Grotesque typefaces used modern Roman proportions, while the stroke contrast was almost entirely omitted. They aimed to be monolinear, which brought a new understanding of typographic form, i.e. it fitted perfectly with the reduced modernists’ forms, which, striving to stylistically ground the discourse, turned to totalitarianism.

**Figure 4.**
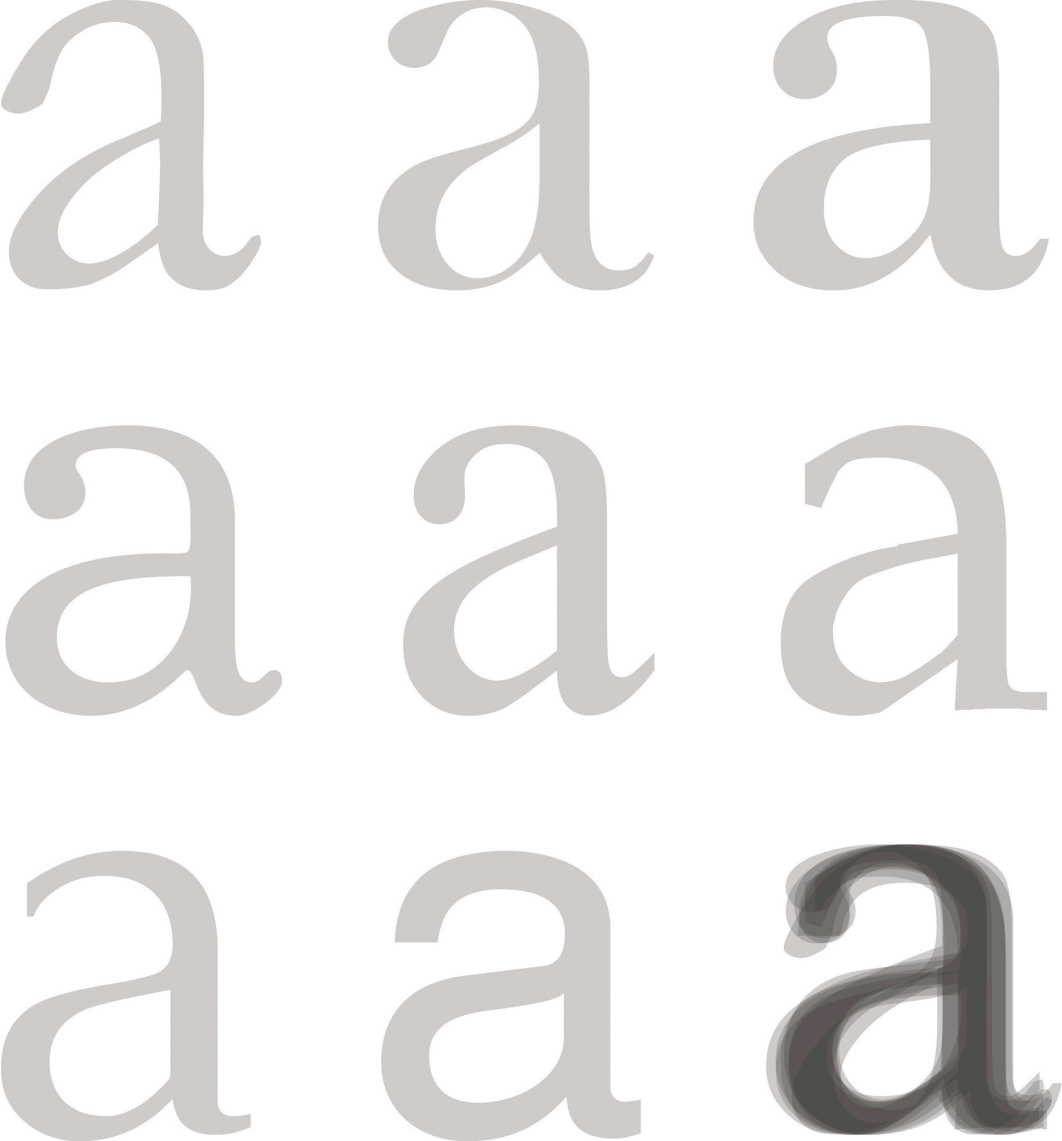
Frutiger’s (8) Common skeleton, adapted.

Looking at the development stages of the late antiquity and early Christian type forms, the turn from majuscule to minuscule, and the joining of the two during the Renaissance, we can see that the shapes and graphemes we know today went through several developmental phases and reforms. With only a few exceptions, the essential structure of the Latin alphabet letters or the “skeleton” as defined by Adrian Frutiger ( 8) has stayed more or less the same for more than 500 years (Figure 4). Frutiger reminded us that the fundamental letterforms have not changed since the Renaissance (9), while, in term of the letter skeleton, only certain letters (‘a’ and ‘g’) show allographic (alternative forms of a letter) variations. All other form variations have to do with style. Their effects, however, and whether they set general or hidden rules, are a subject for an extensive research of a hundred-year interval, which will only give rise to new research questions.

### Gothic and Latin type

Although Humanism touched all areas of Western Europe, the Humanistic type was not widely used. Gothic scripts, especially Fraktur, dominated the German-speaking territories, and then, the German Empire, well into the first half of the 19th century. They could not be replaced despite the attempts of German scholars and academics to reform the script during the Enlightenment, and later, in the mid-19th century.

Gothic scripts were promoted as fundamentally German, especially in situations when the German identity was under pressure (10). The first time that the Gothic script was proudly referred to as national was during the Reformation, more specifically, the printing of Martin Luther’s translation of the Bible, using Schwabacher typeface, in 1523 ( 10).

During the 16th century, Gothic scripts were in use across northern Europe, while Roman and Italic types spread from Italy, across France, to the Netherlands. Sofie Beier (11) notes that in 1539, a Dutch punchcutter, Joos Lambrecht, encountered problems while trying to introduce Roman types to the public. He was ashamed of the uncivilised attitude of the Dutch country people, who were unable to read their own language when printed in Roman type, saying that they did not recognise the letters (12). England and Sweden finally switched from Gothic to Roman typefaces in the first half of the 17th century. According to Burke (10), Germany’s insistence on the Gothic type is actually a consequence of the cultural repression the Germans suffered after Napoleon’s occupation. Thus, the script became a figurative bastion of German values.

Throughout the 19th century, a number of German academics and scholars argued about the irrationality of printing books in Gothic typefaces. Jacob Grimm believed that, in a contemporary context, Gothic letters were unsuitable and ugly. Furthermore, he stated that German books looked “barbarian” in comparison to books of other European countries, printed in Roman. Therefore they damaged their international reputation (10). Consequently, his first edition of German grammar was printed in 1819, using Fraktur. He even made significant orthographic reforms, and also printed the second edition (1822) in Roman type (13). In his study “On legibility of ornamental fonts,” Rudolf von Larish (14) criticised Gothic type as overcomplicated and lacking the distinction between graphemes (a unit of a writing system). Georg Christoph Lichtenberg, on the other hand, stated that German texts printed in Roman type seemed foreign, while Otto von Bismarck claimed that he could read Gothic texts faster than the Roman ones (10).

The final abolition of the Gothic script came not as a result of the avant-garde movements’ actions at the beginning of the 20th century, but strangely albeit logically, in 1941, after the Nazi Germany had already occupied France, Belgium, the Netherlands, Denmark and Norway – countries which had developed the Roman type and were it was widely used. The reason behind this, as the decree addressed to all city councils stated, was to make Nazi message clearer to the residents of all the countries of the Third Reich (10). Apart from that, looking at the Third Reich’s visual rhetoric, we can see that the real motive for this change was propaganda, reflected in the empire’s public architecture, and finally the Roman Empire’s script, which was used to support its authority (15).

### Orthographic reform

At the beginning of the 16th century, when Fraktur was designed by the order of German Emperor Maximilian, it was decided that all nouns should start with a capital letter. The first attempt to reform this rule can be found in Jacob Grimm’s “German Grammar” (“Deutsche Grammatik”) from 1822, where capital letters were used only at the beginning of sentences and in proper nouns. In addition, the book was printed in Roman type, which was a rarity and a sign of resistance in scholarly circles. In his introduction to “The German Dictionary,” from 1854, Grimm argued in favour of this orthography and the abolition of Fraktur, wishing to harmonise German with other European orthographies ( 13).

**Figure 5.**
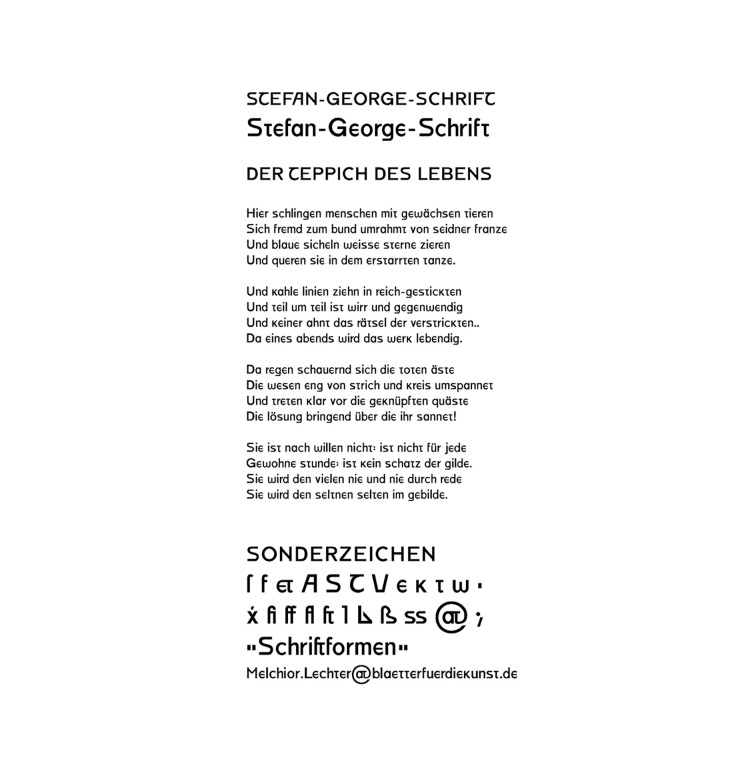
Digitaly reconstructed typeface of Stefan George, Roland Reuß’s (2003) project.

Several decades later, at the very beginning of the 20th century, the use of Grimm’s orthographic reform was recorded. German poet Stefan George asked his friend, Melchior Lechter, to design a typeface based on his sketches, which resembled the half-uncial type. For conterminous letters, Lechter used shapes from Akzidenz Grotesk (16). He also designed a single-storey ‘a’ and uncial forms of ‘e’ and ‘t’ (Figure 5). From 1904 to 1907, this typeface was used for printing Lechter’s works. Since capital letters were present only at the beginnings of sentences and rhyme lines, the support to Grimm’s principles is evident.

A significantly more radical support of the reform came from Walter Porstmann’s book, “Language and Type” (“Sprache und Schrift”), published in Berlin in 1920. Porstmann’s reform included not only the rejection of capitalisation but the modification of some letters, which enabled clearer phonetic transcription. The one who brought Porstmann’s proposals to the Bauhaus was László Moholy-Nagy. The Bauhaus’ liberally cited and interpreted ideas led to a series of experiments among the Moholy-Nagy’s students. Herbert Bayer was the first among them to offer an interpretation of the fundamental idea – the Universal type, presented in 1926, which excluded capital letters.

### Experimental typography in modernism

The beginning of modernism introduced a new dialectical discourse. The new period, which did not rely on the near past or tradition, came five hundred years after the revival i.e. the Renaissance. Therefore, the typographic heritage was brutally treated by some of the early avant-garde artists. This resulted in a need for new principles in modernism which zealously strove towards clarity and the “rhetorically neutral” typography.

Modernism began and developed as a mega culture, a project of modernity which entailed progressive prosperity, evolutionary separation from tradition, and was sometimes even stimulated by revolutionary transformation (17).

There are two natures in modern art, two flows, which are self-determined by the questions of the nature of synchrony and diachrony in art – whether they follow the continuity of the autonomous artistic values or the catastrophic and revolutionary breakthroughs and excesses which change the meanings, concepts, and the art practice itself (as it was done in avant-garde and post-modernity).

Even if the tendencies of modern art and architecture seemed different, all of them preceded and encountered in the point of intersection of one of the historically independent innovations. To start over and to think “ab ovo” is the fundamental and uniting maxim of all movements and kinds of modern art and architecture (18).

Typographical modernity began developing only after the separation from historicism. First came the avant-garde (especially Marinetti’s) with its “bestial” destruction of typographic image and tradition, and then the catalysis of artistic and craft pastiche of the old age in the graphic work of the Wiener Werkstätte at the beginning of 1920s. In the field of graphic design and typography, modernity determines the flow from the source – the avant-garde De Stijl movement, to the Bauhaus’ elementaristic-constructivistic aesthetics and the articulation of the “New Typography” in Swiss typography, i.e. the International Style.

Moholy-Nagy’s constructional style was supplemented by De Stijl influence and postcubic forms from the sculptor workshop of Oskar Schlemmer. Therefore, with his arrival, the elementaristic-constructivistic aesthetic was adopted by the Bauhaus.

The Elementary typography became a new movement, which at first was more of a protest and negation of all that came before, rather than a position based on the foundations of a true theory. In the Elementary typography, the image of the text was radically changed. The central axis was completely abandoned and was replaced with left-aligned text rows of unequal lengths which resulted in a whole new distribution of text on the graphic surface. Decoration and ornaments were proclaimed old-fashioned, and line was recognised as a fully valid design element. The popularity of grotesque typefaces reached its peak around the same time. Among other things, the new typography reopened the debate on the simplification of the German language orthography. Paul Renner, Jan Tschichold and their colleagues from the Bauhaus school were united against Fraktur and other typographical styles derived from handwritten forms, and were advocating sans-serif typefaces and a style adjusted to modern times.

Having finished his studies in 1925, Herbert Bayer became a lecturer and started a printshop at the Bauhaus. Like Moholy-Nagy before him, Bayer also was in favour of the Kleinschreibung doctrine. This system used lowercase letters, as opposed to the conventional, centuries-long method of writing and printing using a combination of lowercase and uppercase. Bayer reserved the uppercase for posters, page designation, and other elements of accidence typography, while the rest was printed in lowercase. This was a rather controversial move, given that all nouns in German nouns are capitalised. At the end of 1925, the page footer of the standardised memorandum and certain Bauhaus publications featured some of the following lines: “We write everything in small letters, thus we save time. Moreover: why two characters when one does the job? Why write big when we cannot pronounce big?”

**Figure 6.**
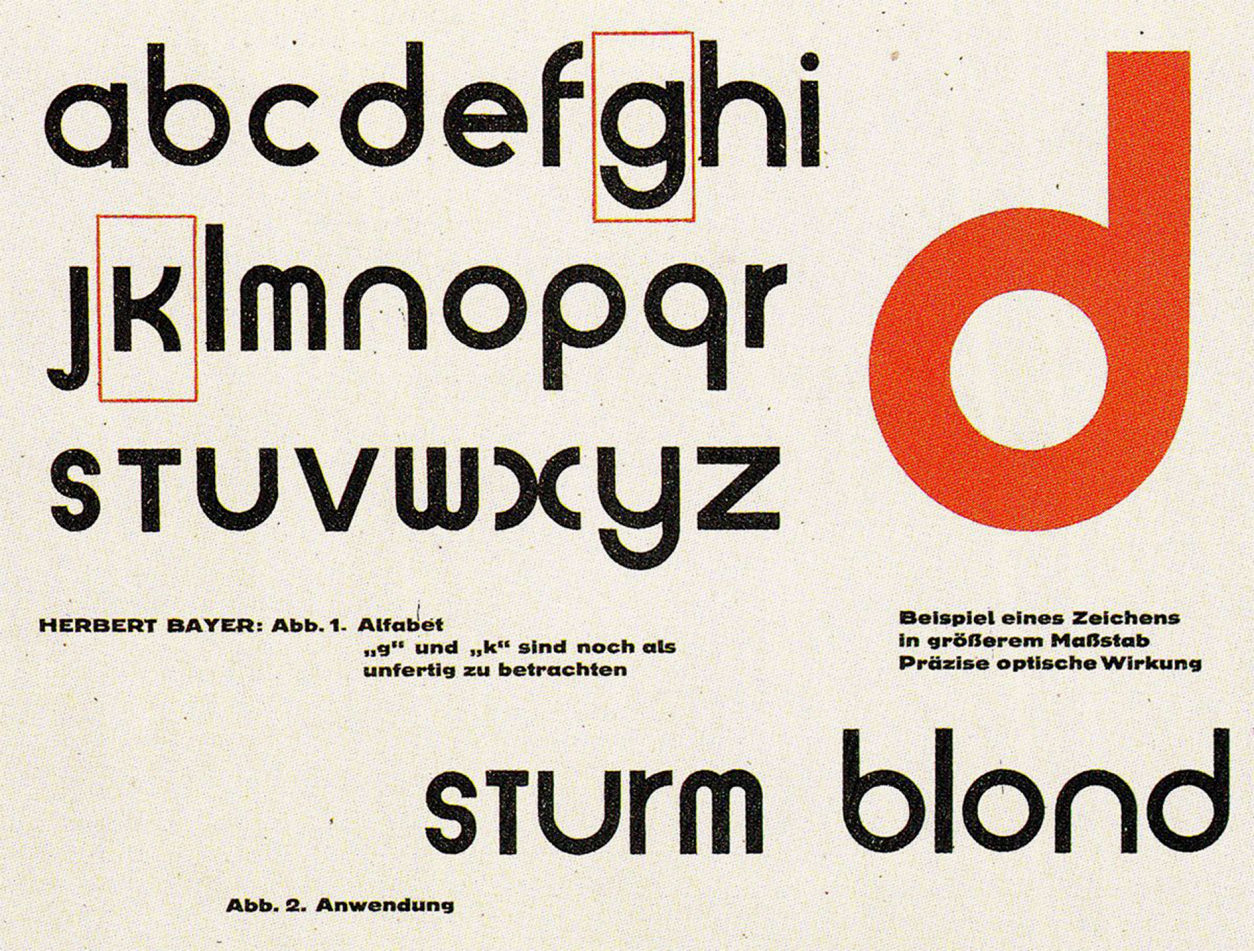
Herbert Bayer’s ‘Universal Type’ (1926).

Given that freeing buildings of all non-functional elements was one of the key principles of the Bauhaus movement, it comes as no surprise that it was applied in typeface design too. Bauhaus proposals strove to relieve the characters of the redundant decorative strokes, and before long, remove the unnecessary capital letters, as well. Suddenly, in 1926, Bayer came out with a new alphabet, presenting the so-called “Universal Type” which featured no capital letters at all (Figure 6). His was a reductive approach, as he kept the lowercase characters and discarded the capitals, finding the latter phonetically unjustifiable. His project aspired toward a solution historically proposed by uncial ( 19).

Even though Jan Tschichold (20) did not ascribe much importance to this radical cut, he supported the orthographic reform as a form of an experiment. In a chapter of his “The New Typography,” which he opened with “Orthography at the present or all in lowercase,” he discussed the problems with bicameral alphabets from both the language and aesthetic standpoint. They are significantly less obvious in some other European languages, such as English which does not capitalise every noun. Tschichold pointed to the fact that the practice of capitalisation of nouns began in the Baroque period, and that Jacob Grimm was against it at the beginning of the 19th century. Many have recognised the aesthetic problem in the mixture of the two very different typefaces. Therefore, many designers preferred using the versals only, avoiding the combination with the lowercase, and vice versa. Tschichold, i.e. New Typography was in favour of a compromise. He was of the opinion that a total alphabetical redesign was unpractical and therefore, an unacceptable solution. According to him, it was possible to both use Bauhaus’ proposals and not to abandon the versals in some special occasions. Since the Kleinschreibung deviated from the fundamental principle of “The New Typography,” when it comes to clarity, Tschichold saw it only as an experiment. Thus the orthographic revolution, an attempt to radicalise the type, was stifled.

Tschichold (20) was, however, more concerned with the choice of a typeface that could best communicate in the new typography idiom. He was looking for typefaces that would be “easily legible; they are also above all in a technical sense useful and free from personal idiosyncrasies – in the best sense of the word: uninteresting.” Tschichold was not satisfied with the available sans-serifs nor with the recently designed ones (Erbar Grotesk, Kabel), finding them “too artistic, too artificial in the old sense, to fulfil what we need today.” Such grounded discourse denied the elementaristic-constructivistic design approach. His idea of “a clear” visual language soon prevailed and exerted considerate influence on international design.

### Postmodernism and typographic deconstruction

Unlike modernism, which was at its core ideological and “full of tense dichotomies,” Suzi Gablik (21) saw postmodernism as eclectic, capable of creating and even stealing from other stylistic and genre forms. According to her, it was a movement that tolerated “insecure and conflicting values.” Keedy (22) believed that the contradiction “to be constant, but always new” was attractive to graphic designers, whose work has thus become “ephemeral.” Therefore, the postmodern age is presented in the form of a critical approach that begins to doubt the “purity” of universal aesthetics. The prevailing iconosphere of the 1980s, and the eclectic poetics of the new wave gave birth to a generation of graphic designers encouraged to pursue a postmodern alternative to their role models, with their subjective and individualistic aesthetics – a modernist alternative with a lowercase ‘m’.

Contemporary graphic design is stylistically vague, which can be seen as a consequence of being brought to life at the end of a revolutionary era, which culminated in deconstructive typography. With the development of Apple Macintosh PCs, designers were granted the freedom to not only shape, but place messages using post-script language and hardware support, without being reviewed by “the system”, thus gaining greater ownership of the content.

Heller (23) points out that advanced technology, combined with experimental activities, had resulted in a new visual language, which has helped to disrupt the “readable” and “clean” one.

**Figure 7.**
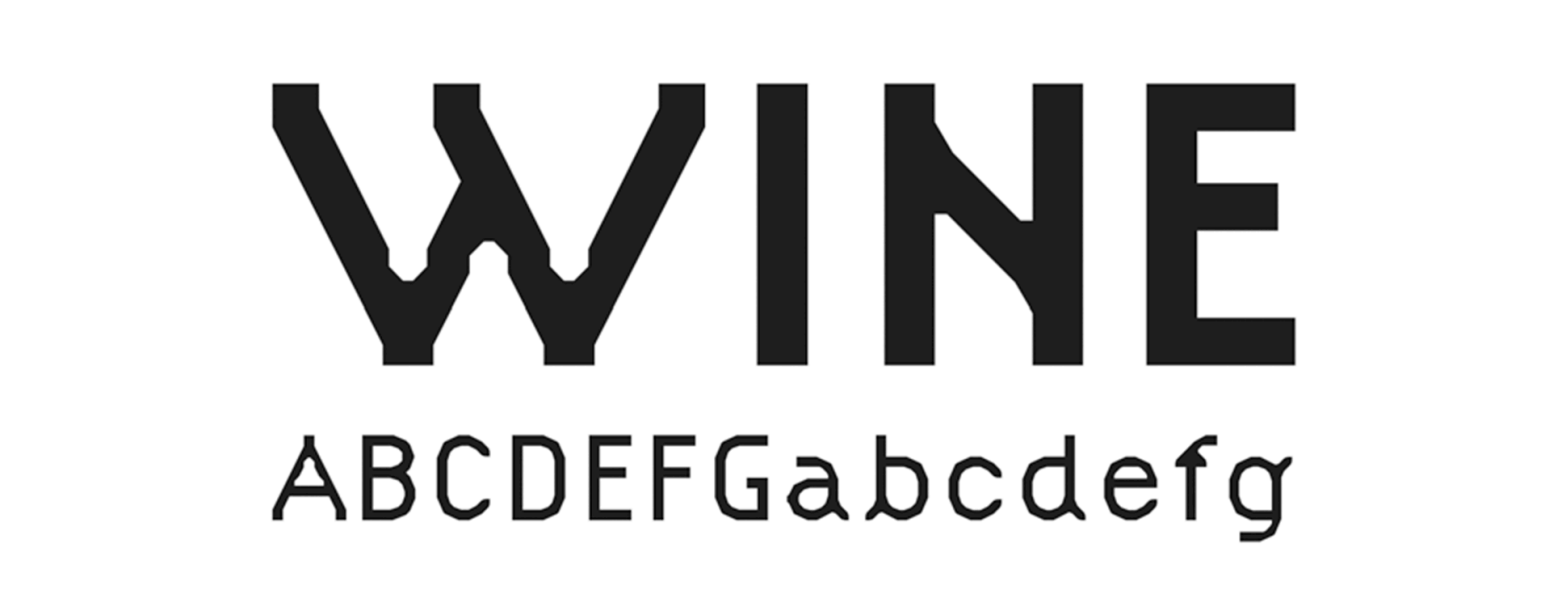
The typeface ‘Citizen’ by Zuzana Licko (1986).

**Figure 8.**
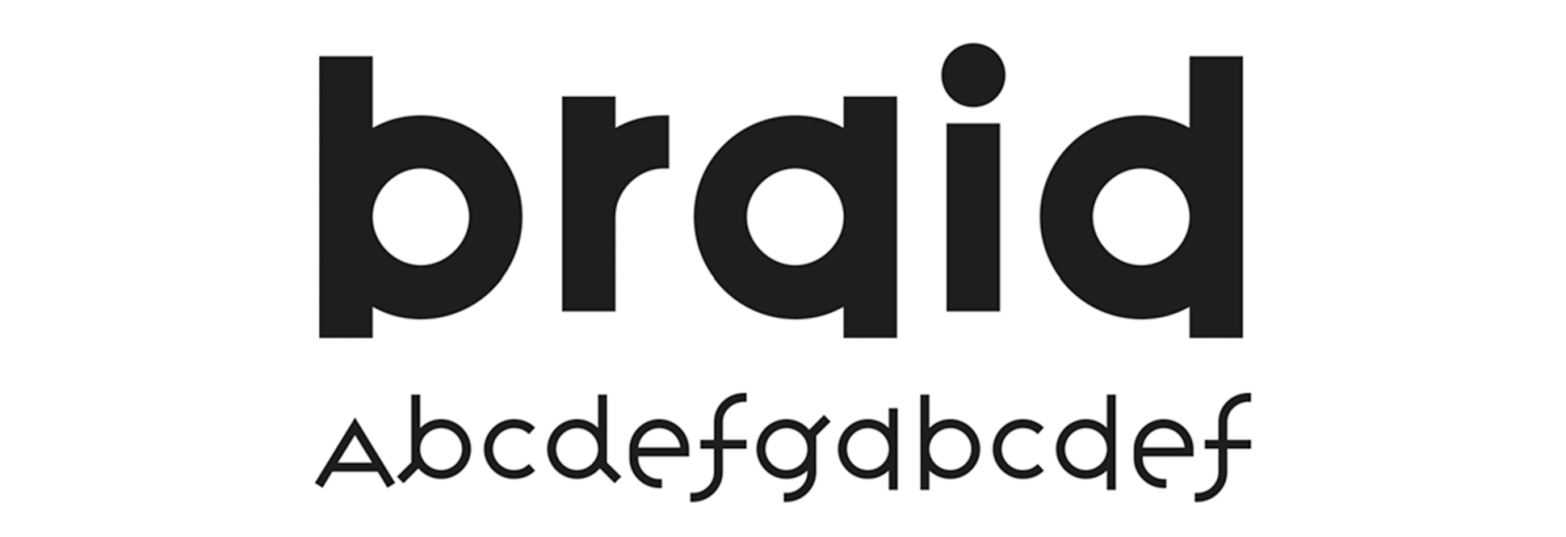
The typeface ‘Variex’ by Zuzana Licko and Rudy Vanderlans (1988).

The Emigre magazine was among the first and certainly the most influential publications of the digital revolution in the desktop publishing. Macintosh appeared on the market in 1985, the same year the third issue of Émigré came out with Zuzana Licko’s first digital bitmap fonts (Figure 7-8).

The reactions to the magazine’s first publications which had been prepared on Macintosh and printed on a low-resolution dot matrix printer, were not good at first. However, some designers immediately recognised the challenge and requested copies of Licko’s fonts. As a result, very soon after, Emigre Digital type foundry was founded (24). This put VanderLans at odds with the critics (modernists) who believed that “the message” has to be legible, and that design in the service of information must be of “neutral” visual character. Emigre’s harshest critic was the veteran of modernism, Massimo Vignelli, who called it “garbage” in one of his articles (25).

**Figure 9.**
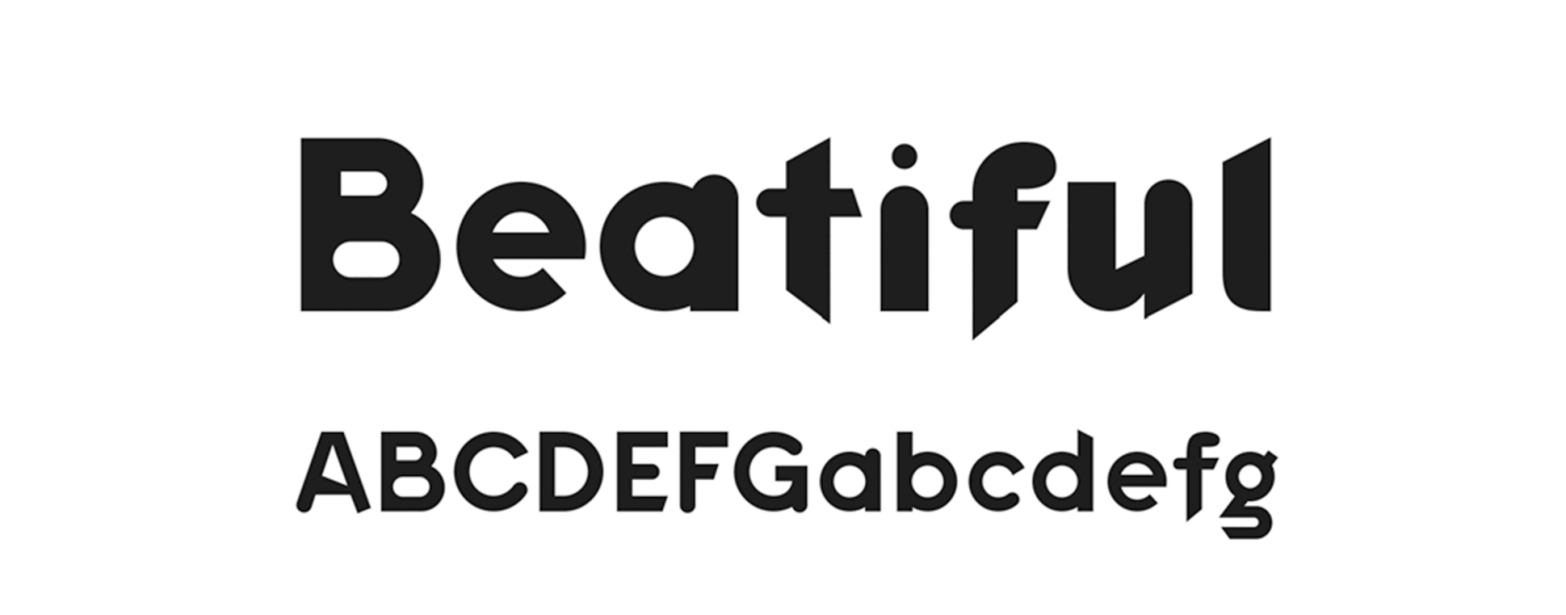
The typeface ‘Keedy Sans’ by Jeffrey Keedy (1989).

Over time, much more advanced graphics applications have been developed that have provided designers with a variety of options. However, the breakthrough in the new digital typography, and its limited capabilities in the hands of the new generation of Rudy VanderLans, the editor and designer of Emigre, inspired a step forward and away from the modernist design principles. Postmodernists believed that there was no absolute truth when it came to typeface legibility. Looking at the issues, Peter Martens (4) saw and pointed to the fact that “letters are what is legible. If something is not legible, then it is not letters. There are no illegible letters. Illegibility does not exist.” Jeffrey Keedy’s belief was that it was enough that conventions on legibility had been established. Keedy (22) did not find his typeface “Keedy Sans” (Figure 9) illegible. Conventions in typographic design, according to Keedy, meant that “everything must be regular. There is always that obsession with regularity and clarity in a simplified way” (5). The most concrete maxim, which, by the way, is empirically tested in this paper, was uttered by type-designer Zuzana Licko (6) in her interview for Emigre – “You read best what you read most.” At the threshold of the digital age, Licko (6) claimed that some typefaces are more legible than others due to their familiarity, i.e. decades or centuries-long exposure to them.

## Empirical review

The numerous legibility studies offer typeface designers of today many relevant findings. Designers in the past, however, were looking at legibility through the prism of answers which confirmed their standpoints and practical results. According to postmodernists, the demand for a legible typeface needlessly restricted creativity, since readers, they believed, have always been able to get used to the new typefaces. Traditionalists and modernists nurtured practices based on to the results of legibility studies from the beginning of the 20th century, and finally, adopted those that supported their stances and discourses, even when they were contrary to the results of numerous other studies. Sometimes they even instructed the scholars cf. ( 26, 27, 28).

Although theoreticians and practitioners had been intensively debating whether serifs affected legibility or not, not even empirical research could give them an answer. The results of various experiments showed that when it came to legibility, there is no difference between serif and sans-serif typefaces (29, 30, 31). Many scholars believed that the findings that support one or the other side were not be externally valid (cf. (32, 33) cited by Lund, 1999), as they noticed the existence of vast differences within both serif and sans-serif groups. Ole Lund (26) found the presence and absence of serifs a possible important legibility factor, but too elusive to measure in the reading process. Many other factors were recognised as the ones having a significant influence on the reading process, such as font size, line length, leading, the overall layout uniformity and the relation between the colour of the text and its backgroun (34, 35), x-height, stroke-width and inner white space (29, 30, 36, 37, 38).

Although a lot of studies had been conducted in this field at the first half of the last century, not many things have actually been established. Many of the studies carried out by psychologists are considered invalid because the researchers lacked typographical knowledge (26). On the other hand, type theoreticians have come up with numerous unfounded claims, trying to stylistically and ideologically establish their discourses (39).

We can conclude, nevertheless, having reviewed and compared the various findings, that in the field of typeface legibility, certain fundamental principles do exist, and that they are based on differentiation (40, 41) and familiarity (2). Although not recognised as actual typographical theoretic knowledge, those principles can be found in the epistemological studies of Dirk Wendt (42), Ole Lund (26) and Sofie Beier (11). One of the fundamental principles analysed in detail by Nedeljković et al. (41) is letter differentiation. This means that in order to recognise a letter a person has to be able to first notice its particular features during the inductive information-processing, first of all the terminals (43). In addition, we should mention the findings of Sofie Beier and Kevin Larson (40), which allow us to determine those stylistic attributes that can affect letter recognition in terms of distance reading. On the other hand, there are two noteworthy studies on familiarity, which concern Frutiger’s skeleton. The study by Nedeljković et al. (44) examined the relationship between typefaces’ personality attributes and their congruence with universal structure. Beier et al. ( 45) examined the legibility of embellished display typefaces, which on different levels relied on Frutiger’s common letter skeleton.

Tomas Sanocki (46) conducted an experiment in which he had several strings of letters, and where one string consisted of letters of the same font, and the other of a mix of two. He found that reading accuracy was greater when a person was looking at the same-font string then the two-font one. He concluded that “perceptual system becomes tuned to the regularities of a particular font in order to process visual information efficiently” (46). This is one of the first studies that confirmed the presence of the font tuning phenomenon. Font tuning studies focus on factors that enable recognition and memorisation of structural regularities, in order to make text perception faster and more effective.

The font tuning effect in a way proved Licko’s (6) claim that “we read best what we read most.” With it, however, she promoted deviation from the traditional and “modern” approach in typeface design, which is glued to the “prototypical” or “universal skeleton.” What she actually meant was that the legibility of all known typefaces is the result of their long-lasting and frequent usage. Therefore, all new non-prototypical typefaces (she designed), would be as legible as Times or Helvetica are today, only if they were to be used as frequently. This, nonetheless, has not yet been proven, even with the results of the Beier and Larson (47) study, which will be explained in more detail in the following sections.

### Theory and hypotheses development

Numerous studies have supported the thesis that text set in a consistent and regular font is easier to read than the one in which font properties vary (35, 48, 49, 50, 51, 52), e.g. uppercase and lowercase are mixed. The effects of regularity in these studies were examined at a level of word recognition. Corcoran and Rouse (49) noted poorer identification levels in cases when the font format changed from word to word. Klitz et al. ( 52) observed that paragraphs set in mixed fonts took more time to read. The theory that the human perceptual system actually adapts to a typeface, however, has been proven by a few studies (1, 3, 47, 53). Thomas Sanocki gave a significant contribution to this field, by publishing a series of studies (1, 54, 55), in which he explained the phenomenon of letter structure and how the visual system reacts to that structure, by varying different influential factors. Sanocki started with an assumption that a reader, when faced with a particular font for the first time, notices the structural characteristics of the letterforms, which he then commits in his long-term memory. The next time he encounters the same font, he draws the information from memory, thus accelerating the reading process. This process is based on the principle of recognition of general features (such as spatial frequency and font size), which constrains the search through the perimeter space, consequently allowing the search for a concrete structure – local font characteristics (1, 54). This effect is called the font tuning effect.

The most recent study in this field aimed to answer how typeface familiarity influences reading speed and readers’ preferences (47). It has tested two hypotheses: the prototype hypothesis and the exposure hypothesis. The former is based on Frutiger’s common skeleton (8) and refers to the similarity between letter shapes and structure, which in a unique way clump together the same letterforms of different typefaces. The latter is based on the level of familiarity with a particular font, i.e. the ability to quickly match attributes – tuning. Although Beier and Larson had created new typefaces for their study, they did not succeed in proving their premises.

The goal of this study is to see if recognition of a new typeface comes from experience, i.e. typeface familiarity. Thus, for the problem of the unknown typeface to arise, the reader must compare it to the known: typeface, style and structure. Therefore, in this legibility study, the effect of familiarity is tested. The precise research question is: Does the font tuning effect depend on a typeface’s similarity to Frutiger’s skeleton or not?

We began with an assumption that a reader would read a text set in a familiar typeface under repeated conditions with equal efficiency. If the reader is, however, exposed to a new font over a set period of type, his/her visual system would successfully identify font regularities. That would result in faster text processing during the next encounter.

This study aims to prove that font tuning depends not only on the exposure period, but also on the universality of the skeleton’s structure, and formulates two hypotheses.

H1: Exposure to a typeface provides better legibility.

H2: Exposure to a typeface and the universal skeleton provides better legibility.

We compared reading performances at different familiarity levels (dependent on the exposure and common-skeleton similarity).

The experiment aims to examine the established hypotheses, i.e. to check whether human visual apparatus accommodates to the universal characteristics of a typeface and if the time it takes to font tune depends on the reader’s familiarity with the type.

To test the hypotheses we needed to test the influence of familiarity by using three different typefaces and check whether typeface familiarity, and the overall reading comfort, is a result of the exposure period, the common skeleton or both.

The expected result for hypothesis H1 is that the reading speed of the known-common typeface would remain unchanged after the exposure period. Additionally, the reading speed of unknown typefaces would increase after the exposure period. The hypothesis H2 would be proven if it turned out that the reading speed of an unknown-common typeface is higher than the reading speed of an unknown-uncommon typeface.

## Methods

### Design of stimuli

For a study which examines the influence of a typeface upon legibility, the ideal stimulus material would be a “custom made” set of typefaces, i.e. experiment under completely controlled conditions, where only the parameters to be tested vary in a predetermined way, maintaining all other variables constant (42).

By analysing the results of Beier and Larson (47) study, we came to a conclusion that a possible reason the study was unsuccessful in proving the prototype hypothesis is the inadequacy of the used stimuli. The tests were done using new typefaces which, when it came to a few letters, relied on uncial characters and kept the versals. This meant that certain letters strayed from the Frutiger’s skeleton. Graphemes of lowercase letters ‘n’ and ‘t,’ for instance, were in the form of small capitals. The authors also made stylistic alternations of the graphemes ‘a’ and ‘s,’ whereas the lowercase ‘a’ was given a distinctive hook at the top, and the lowercase ‘s’ a distinctive finial. Since there were only four characters that did not match with the prototype skeleton, the study could have yielded different results had more characters been altered. To back this, by explaining the aspects of the final familiarity form, Beier and Larson (47) recalled the examples of the alternative forms like those of Herbert Bayer and Wim Crouwel. Even though these typefaces are unicase, typeface Grid Sans Unicase has been designed for the purpose of this study.

Therefore, Grid Sans Unicase was conceived as a version of the modernist uncial, i.e. a unicase typeface based on Peignot which had been designed by A.M. Cassandre.

In addition, the likely reason for their obstacle to prove the exposure hypothesis could be that Beier and Larson (47) compared typefaces of very similar structure in both groups. The new typefaces, Pyke and Spencer, created for the experiments, both have a bookish font structure, humanistic skeleton and are very elegant. Furthermore, they share stylistic and shape attributes with many well-known typefaces (such as Helvetica and Times, which were used as control stimuli), the only difference being the shape of terminals and serifs, as well as pseudo serifs (typeface Spencer). In the first phase of the trial, the designed stimuli were read equally fast or even faster than the control stimuli. What was unexpected was that when the tests were repeated, the results stayed the same, which means that essentially those typefaces were not new to the readers.

Given that this study wants to test the same hypotheses, we used Grid Sans, which was designed to have as many as uncharacteristic attributes as possible. At the same time, it does not deviate from the humanistic prototype, i.e. the universal skeleton.

**Figure 10.**
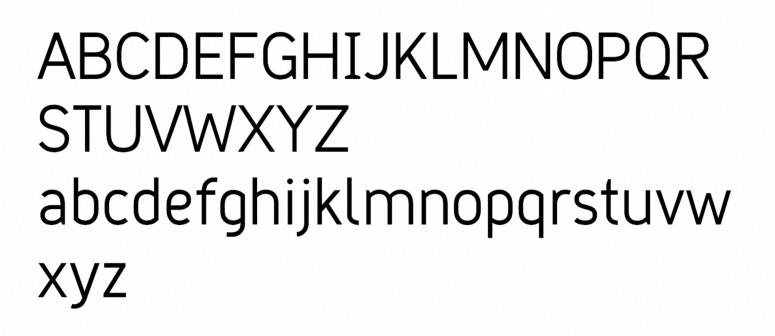
The typeface ‘Grid Sans.’

**Figure 11.**
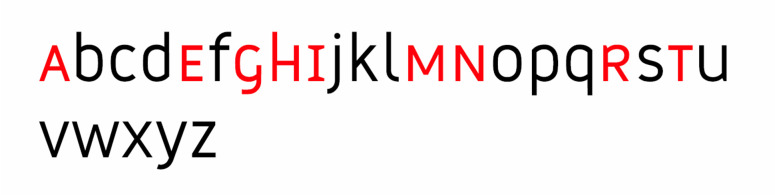
The typeface ‘Grid Sans Unicase.’

The typeface Grid Sans is monolinear and is emphatically grid-constructed. Therefore, according to its formal attributes, it can be classified as a kind of Geometric Grotesque. Nevertheless, unlike other typefaces from this group, Grid Sans does not have typical allograph of the lowercase ‘a’ or the classical proportions which were tied to either a circle or rectangle. The monolinear feature of the typeface is supported by the purposely shortened stems at junction points with arc strokes while being discretely abandoned at the junctions themselves. Aside from this, Grid Sans’ ‘e’ has a unique eye (Figure 10-11). The reason behind this was to have the reader recognise the typeface as new, according to the aim of the study.

When designing a font, metrics adjustment is equally important as individual characters design. Every grapheme consists of a black form and white antiform. Changes in those ratios consequently change the visual rhythm. Many type practitioners (e.g. (56, 57, 58, 59)) wrote down their experiences, and thus defined the instructions for the best practice of type metrics, i.e. the instructions for right and left side bearing for every letter (60).

In order to define the metrics for our typographic stimuli, we did pretesting. We used and compared three methods in spacing typeface Grid Sans (61): Walter Tracy’s (58), Miguel Sousa’s (59) and the automatic metrics adjustment of the FontLab Studio 5.2.1. application. The results showed that the automatic metrics is rigid and does not provide satisfying results, while the best results are obtained through a combination of Tracy’s and Sousa’s methods.

Font hinting represents the last stage of font design, and it provides instructions for rasterisation, i.e. rasterising the characters on the output device. The stimuli were generated using the FontLab Studio 5.2.1. software for Windows OS.

### Testing stimuli

The experiment, as mentioned earlier, was carried out using three typefaces, each representing one level of familiarity, based on novelty and congruency with the universal skeleton. Those levels are described in Table 1.

**Table 1:** Different familiarity levels of examined typefaces.

Familiarity level		Group’s representative
Known	High level of skeleton structure regularity (common skeleton)	Arial
Unknown	High level of skeleton structure regularity (common skeleton)	Grid Sans
Unknown	Low level of skeleton structure regularity (uncommon skeleton)	Grid Sans Unicase

The first typographical stimulus is from the group of typefaces whose familiarity has already been developed because of their wide-spread use, long-lasting exposure and universal skeleton structure. As a conventional representative, we chose to use the Arial typeface. Helvetica and Times New Roman are also parts of this group.

**Figure 12.**
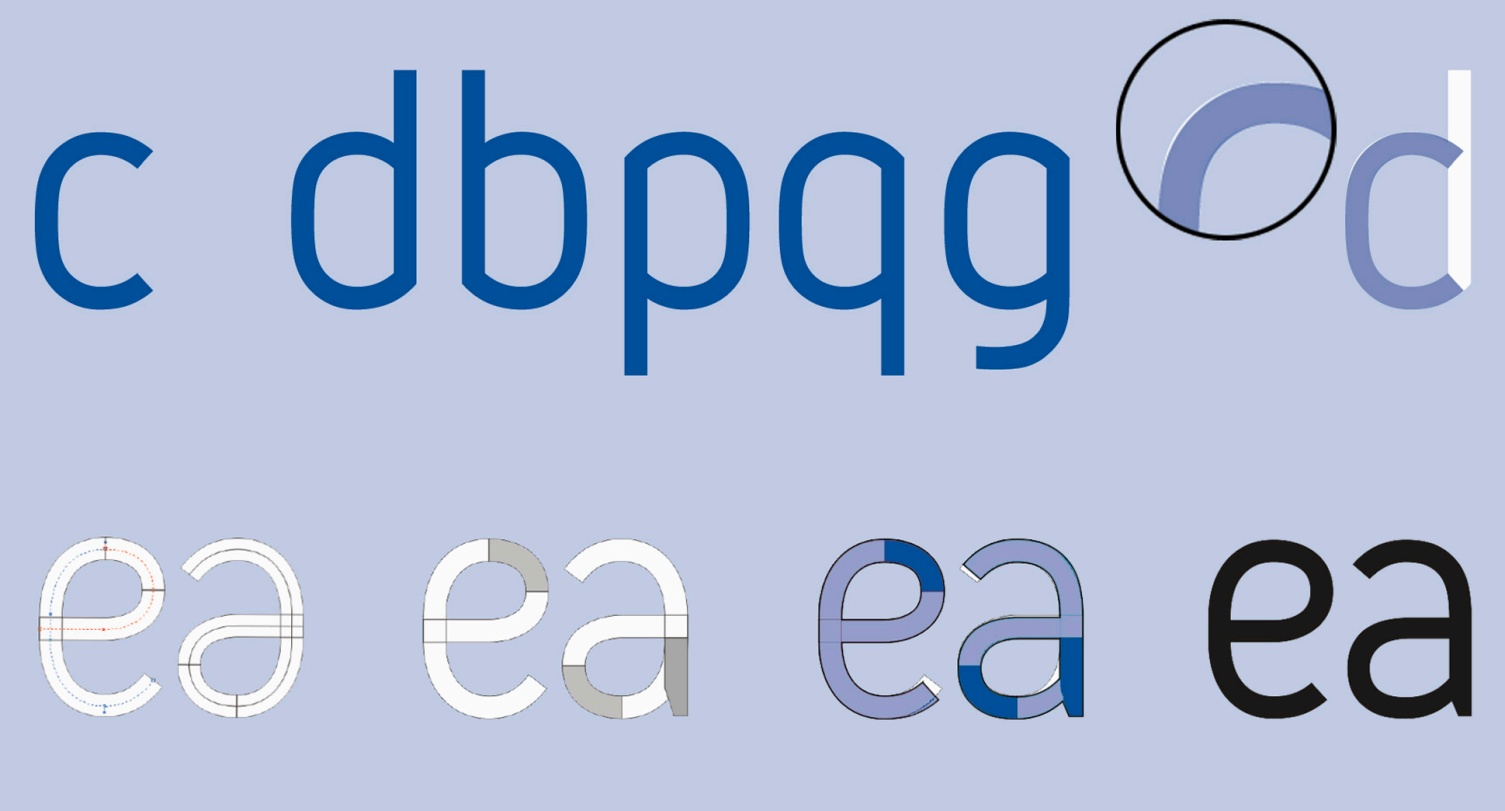
Specific attributes of the typeface ‘Grid Sans.’

The second stimulus, Grid Sans, is one of the newly designed typefaces, previously undistributed. Its design is based on the universal skeleton, which, as we presume, provides some level of familiarity. Apart from the skeleton, Grid Sans shares the geometry with Arial but differs in specific attributes: width, characters’ openness, lightness, junctions, and special characters (Figure 12).

The third stimulus, Grid Sans Unicase, is a new, undistributed typeface with unconventional graphical characteristics. This typeface follows the avant-garde principle of Herbart Bayer and early typographical experiments of Rener, Tschihold, Belmer, Bill and A. M. Cassandre.

**Figure 13.**
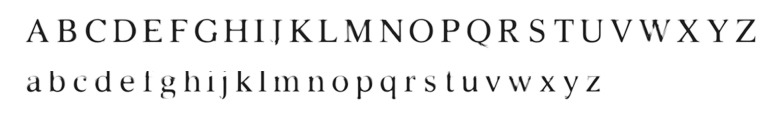
Humanistic skeleton prototype.

The level of congruency of stimuli with the humanistic prototype is examined using digital picture analysis, i.e. by analysing the binary matrix using ImageJ software. The shape of the humanistic prototype is the result of median values of eight typefaces (Garamond, Baskerville, Bodoni, Excelsior, Times, Palatino, Optima and Helvetica), which Adrian Frutiger (8) used to define its skeleton. The median of all samples is obtained by their overlapping in the same-resolution matrix, where all samples are aligned vertically and to the baseline. The result is a picture of the median value of every pixel (Figure 13). Step-by-step instructions are given in the study of Nedeljković, Novaković, & Pinćjer (44).

Every letter of the stimuli (Arial, Grid Sans and Grid Sans Unicase) was compared to its same matrix resolution prototype pair using the structural similarity index (SSIM). The results of the analysis are similarity indexes for each grapheme with a defined prototype where “index value of 1 represents that two images match 100 %. As the index value falls closer to 0, the difference between the two compared images is bigger” (44).

Table 2 shows the descriptive statistics for the examined stimuli. To determine the matching level between the stimuli and the prototype, we performed ANOVA test (*p*=.003). Tukey’s HSD Post Hoc test shows that Grid Sans Unicase (unknown-uncommon) differs significantly from both Arial (known-common) on the level *p*<0.05, and as Grid Sans (unknown-common) with *p*<0.01 (Table 3). At the same time, Post hoc test does not show a significant difference between Grid Sans and Arial, which qualifies all three typefaces as appropriate stimuli for our experiment.

**Table 2:** Descriptive statistics of SSIM to prototype.

Typeface	Mean	Std. Deviation	N
Arial	.8514087	.03878200	52
Grid Sans	.8564638	.02809938	52
Grid Sans Unicase	.8331883	.04060628	52

**Table 3:** Tukey’s Post Hoc test results.

(I) font	(J) font	Mean Difference (I-J)	Std. Error	Sig.	95% Confidence Interval	
					Lower Bound	Upper Bound
Arial	Grid Sans	-.0050552	.00710948	.757	-.0218815	.0117711
	Grid Sans Unicase	.0182204*	.00710948	.030	.0013941	.0350467
Grid Sans	Arial	.0050552	.00710948	.757	-.0117711	.0218815
	Grid Sans Unicase	.0232756*	.00710948	.004	.0064492	.0401019
Grid Sans Unicase	Arial	-.0182204*	.00710948	.030	-.0350467	-.0013941
	Grid Sans	-.0232756*	.00710948	.004	-.0401019	-.0064492

### Participants

Eighty-four participants (55 female, 29 male), aged 18-35, voluntarily took part in this study. The group comprised undergraduate students and teaching assistants at the Faculty of Natural Sciences and Engineering, the University of Ljubljana, whose native language is Slovenian (the official language in Slovenia). All participants had either normal or corrected-to-normal vision.

Each participant was assigned to one of three conditions upon entering the lab. They were all unaware of the hypothesis and were only told to read as naturally as possible. Before the beginning of each session, every participant was orally informed about the next steps.

One data set per group was eliminated from analysis as an outlier.

### Procedure and materials

**Figure 14.**
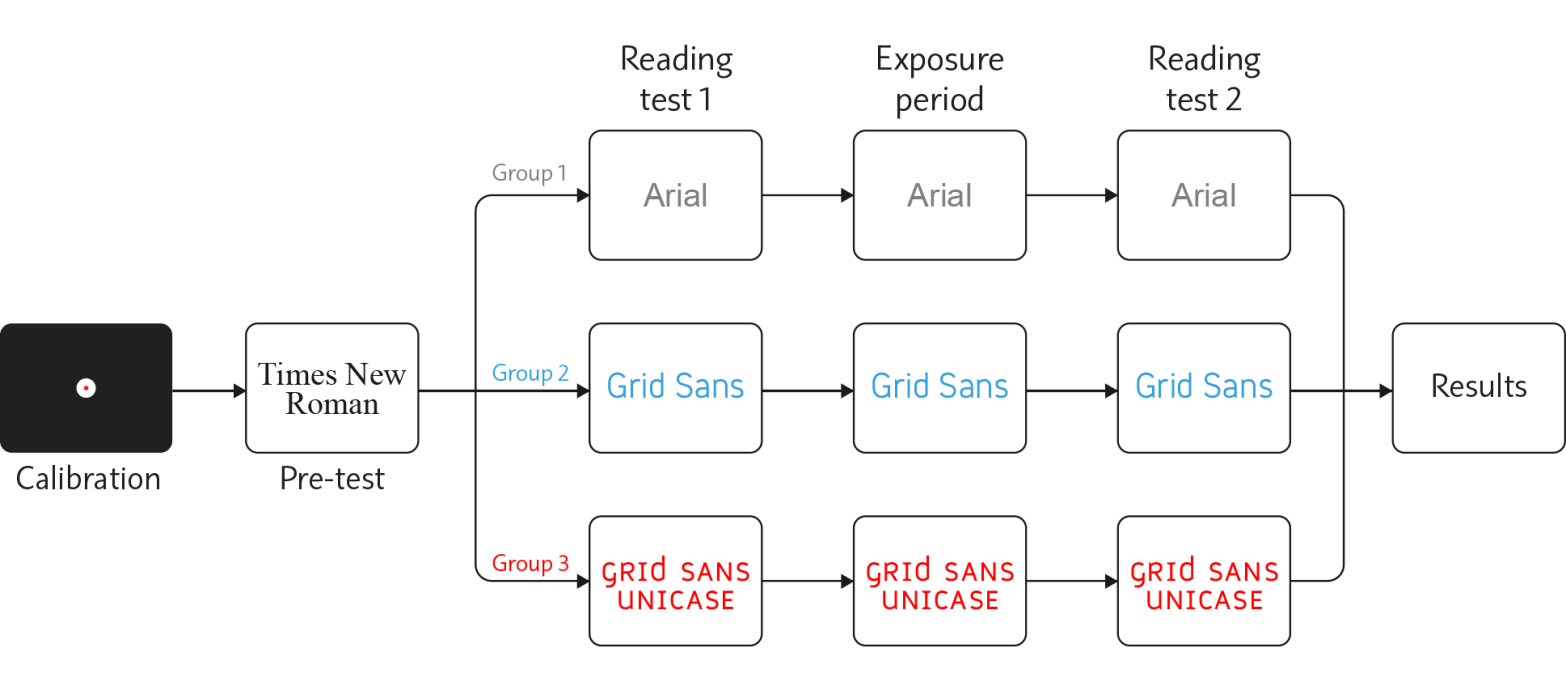
Experiment procedure.

Each participant underwent a single experimental session. Each test session consisted of five stages: calibration, pre-test reading, reading speed test 1, 10-minute exposure period, and reading speed test 2. Figure 14 schematically describes the order of the stages.

The variables under scrutiny were isolated in order to alter one visual feature at a time, i.e. all other text parameters were constant, so the only difference between the treatment groups was the typeface. We chose four texts and set them in different typefaces, so each participant read the same content.

The text for the reading speed test 1 contained 156 words, i.e. 647 characters excluding spaces. The text for the reading speed test 2 contained 148 words, i.e. 656 characters excluding spaces. The passages were written in Slovenian language, and were excerpts from Ela Peroci’s children book “For good night.” The texts used for testing the exposure period were copied from a Slovenian lifestyle magazine and were about popular music, film and technology.

The paragraphs were 588pt wide, justified, with the last line left-aligned. The x-height of every typeface was the same, so we used different font between groups. The texts in Arial were set in 28.25pt font size with 33.9pt leading while the texts in Grid Sans and Grid Sans Unicase were set in 26pt font size and 31.2pt leading. The colour of the text was black, and the background was white.

The experiment took place in a room with neutral grey walls, reflectivity max. 60%, according to ISO 3664 (2015). For the recording of eye movements, eye tracking device Tobii X120 and accompanying software Tobii Studio 3.1.3 were used. The participants were sitting in a comfortable and adjustable chair. The distance from the participants’ eyes to the screen was approximately 65 cm. Five-point calibration was performed.

**Figure 15.**
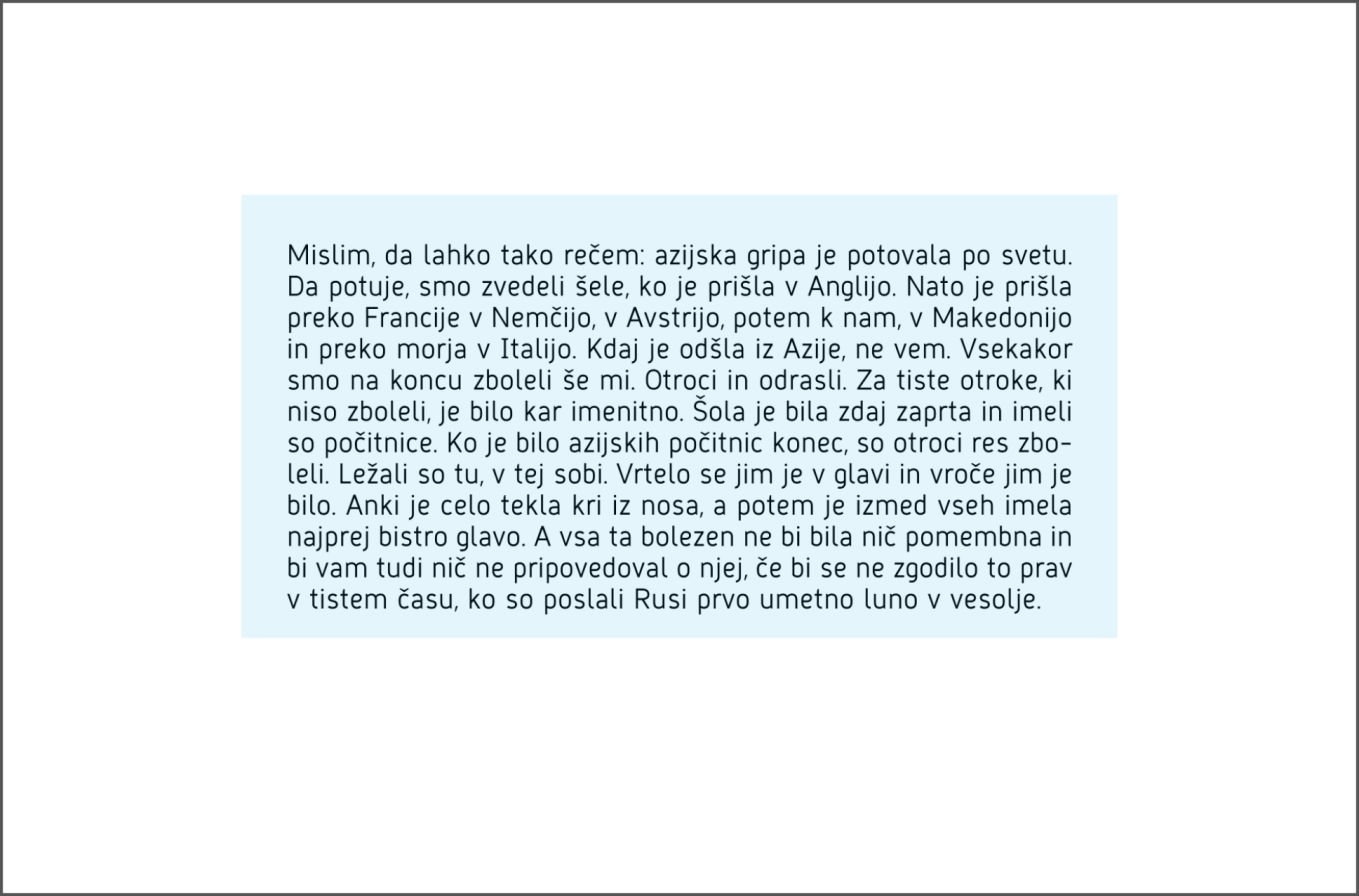
An example of defined ROI.

We drew a rectangular region of interest (ROI) around the texts (Figure 15), in order to obtain the measure of total fixation duration [sec]. We collected the data from the reading speed test 1 and the reading speed test 2, but not from the pre-test reading stage and exposure period since those stages are carried out as adaptive periods.

## Results

Table 4 shows the descriptive statistics for the performed tests. Our aim being to detect the differences in reading speed before and after the exposure period for each treatment group, we used Wilcoxon’s signed-rank test. The significance level (α) was set to 95%.

In the first – control group (known-common typeface) the test did not show a significant difference (p>0.05) between fixation duration before and after the exposure period.

The same happened with the second group (unknown-common typeface), as the test also did not show any significant differences (p>0.05).

The third group (unknown-uncommon typeface) showed a significant difference after the exposure period (p=0.009, r=0.5), with large-to-medium effect size.

## Discussion

The goal of this study was to examine the nature of typeface familiarity. Having reviewed the literature, we looked at typeface familiarity through the lens of typeface novelty and prototype skeleton commonness. Therefore, we concluded we needed new typefaces, one of common and one of uncommon structure, which we could compare with the familiar one.

By including the findings of font tuning studies, we put forward our research question: does font tuning depend on familiarity with typefaces’ common skeleton or rather specific typeface characteristics.

**Table 4:** Reading tests descriptive statistics.

Typeface	Reading test	N	Mean	Std. Deviation	Minimum	Maximum	Percentiles		
							25th	50th (Median)	75th
Arial	1	27	36.0910	12.28622	19.15	81.60	28.8820	34.2700	39.5400
	2	27	35.5707	14.50781	14.99	75.20	25.5300	32.5000	39.7760
Grid Sans	1	27	33.7286	8.37382	15.59	49.57	28.7600	34.1600	37.5700
	2	27	32.9433	10.34225	14.33	62.98	26.3700	33.1600	39.0090
Grid Sans Unicase	1	27	37.3284	9.57049	19.96	58.82	32.1400	37.8950	42.6900
	2	27	34.7413	8.79051	18.91	51.39	29.1190	35.3340	40.0900

Firstly, we assumed that a reader would read the text set in a known-common typeface in the repeated conditions with equal efficiency. The results for hypothesis H1 were as expected since the results of repeated measures after the exposure period for Arial have not changed significantly. This typeface is familiar to its readers given it is a systematic font of the Microsoft Windows platform, as well as the most usual substitute for sans-serif typefaces which cannot be found in standard OS. As such, Arial has been present for three decades. Therefore, we confirmed that typeface familiarity influences the reading speed in general.

Secondly, we hypothesised that if a reader was exposed to a new font for some amount of time, his/her visual system would successfully identify font regularities, which would result in faster processing during the next encounter.

While the results of the reading speed test for texts set in common typefaces (Arial and Grid Sans) did not vary significantly, the same tests run for unknown-uncommon (Grid Sans Unicase) showed noticeably higher reading ease after the exposure period.

The reading speed of unknown-common typeface did not change significantly after the exposure period. Based on the results, we conclude that the universal structure is the constant, which provides reading comfort. Its presence enables legibility of every new typeface form that is derived from the universal skeleton regardless of its specific stylistic attributes. In other words, typeface legibility depends on how much the typeface resembles the structure we had been exposed to.

Why is that so? Do specific characteristics of typefaces affect not only recognition but also motivation and will (62, 63)? Can one’s familiarity with stimuli i.c., typefaces be assigned its value (64, 65)? It is known that many attributes, e.g. cheap, can be ascribed to typefaces according to their specific characteristics (44). The preferences for typefaces exist by habit (6). Suppose we observe the reading habit as a value. In this case, the reader is not familiar with the specific characteristics of a typeface during the early exposure period. Therefore, the typeface does not have a value per se but acquires it over time.

The results of this study confirm that the universal letterform, the one Adrian Frutiger recognised as the prototype skeleton, is what enables familiarity. The fact that the Roman types were so widely used across the Western world led to a general familiarity with the Humanistic form, a structure we have been exposed to over half a millennium. Therefore, practitioners interested in the relationship between typeface construction and legibility should consider the commonness of the humanistic skeleton when creating or choosing typefaces.

This study also examined the legibility of typefaces that are not deemed universal. According to the results of reading an unknown-uncommon typeface, we can conclude that uncommon letterforms are a priori not as legible as the common ones. The legibility of such type forms can be improved by the exposure period, which the experiment results showed. For, we know that the exposure period has a positive effect on legibility, whether or not the typeface matches the universal skeleton.

The differences in the reading speed of the uncommon compared to the common skeleton supports our hypotheses which state that in addition to the exposing period, similarity with the universal skeleton contributes to the familiarity effect, which reading speed depends on.

Our experiment relied on the familiarity study of Beier and Larson ( 47). While testing the same hypotheses, our methodology approach bridges the gap in their study, with stimuli design in the first place. In our study, the newly designed stimulus of the uncommon skeleton (Grid Sans Unicase) differs from the other stimuli, which was confirmed by the SSIM statistical test results, when this typeface was compared to the prototype. Unlike Beier and Larson (47), we have included the minuscule and majuscule differences of the stimuli. In other words, this typeface contains more graphemes that do not match with the universal skeleton i. e. the humanistic prototype. Therefore, Beier and Larson attempt to prove hypotheses could succeed if their typeface varied in the measure our does. Undoubtedly, their study is admirably established, which our success in proving hypotheses validates. On the other hand, the SSIM analysis showed that the other newly designed typeface does not deviate from the prototypical skeleton, although it includes many special characteristics.

Grid Sans Unicase is a form of “modernistic” uncial, orthographically dissimilar typeface grounded in the concept of the modernistic orthographic revolution.

Our study confirms Tschichold’s view that the radical cut of the Kleinschreibung was not in the service of clarity. We can also argue that Licko’s famous claim is proven if we take into account the long period of familiarity with the Humanistic type, which has been in continuous use since its creation.

The results of this study contribute to the field of typography. Further analysis of typeface legibility and familiarity may benefit from our findings, as well as our methodological approach. Our method has advanced the traditional approach in legibility research, since we measured legibility in relation to the total fixation duration on the ROI, using eye tracking technology.

## Ethics and Conflict of Interest

The authors declare that the contents of the article are in agreement with the ethics described in http://biblio.unibe.ch/portale/elibrary/BOP/jemr/ethics.html and that there is no conflict of interest regarding the publication of this paper.

## Acknowledgements

This research was supported by the CEEPUS projects CIII-RS-0704-06-1718-M-117770 and CIII-RS-1205-02-1819(Umbrella)-M-124733. This work was supported by the Ministry of Education, Science and Technological Development of the Republic of Serbia through the project No.:451-03-68/2020-14/200156: ‘Innovative scientific and artistic research from the FTS (activity) domain.’
